# Molecular detection of *Bartonella* species in rodents from the Lao PDR

**DOI:** 10.1111/j.1469-0691.2008.02177.x

**Published:** 2009-04-03

**Authors:** E Angelakis, K Khamphoukeo, D Grice, P N Newton, V Roux, K Aplin, D Raoult, J M Rolain

**Affiliations:** 1 Université de la Méditerranée, URMITE UMR 6236, CNRS-IRD, Faculté de Médecine et de Pharmacie, 27 Bd Jean Moulin, 13385 Marseille cedex 05, France; 2 NAFRI, Ministry of Agriculture and Forestry, Government of Lao PDR, Vientiane, Lao PDR; 3 CSIRO Sustainable Ecosystems, Canberra, Australia; 4 Wellcome Trust-Mahosot Hospital-Oxford Tropical Medicine Research Collaboration, Microbiology Laboratory, Mahosot Hospital, Vientiane, Lao PDR; 5 Centre for Tropical Medicine, Churchill Hospital, University of Oxford, UK; 6 Australian National Wildlife Collection, CSIRO Sustainable Ecosystems, Canberra, Australia

## Introduction

*Bartonella* are gram-negative bacteria classified within the alpha*-Proteobacteria*. Over the last decade the number of identified *Bartonella* spp. has increased rapidly and the known diversity of the Bartonellaceae family continues to expand, as recently demonstrated in various mammals 1,2, including humans 3. The geographical distribution of *Bartonella* species depends on their hosts and vectors. Mammalian species, such as cats, dogs, rodents and ruminants, are the main bartonellae reservoirs. Our objective was to detect and identify, by molecular methods, the presence of *Bartonella* species in wild rodents from the Lao PDR (Laos), a country where epidemiological and clinical studies of zoonoses are scarce.

## Methods

### Mammal samples

Rodents were trapped using cage and break-back traps and purchased from local markets in June and December 2006 in four Lao provinces. All specimens were photographed, vouchered (specimens will be registered in the Australian National Wildlife Collection, CSIRO Sustainable Ecosystems, Canberra, Australia) and identified subsequently (by KA) 4. Their livers and spleens were stored in ethanol prior to laboratory analysis for the detection of *Bartonella*.

### Molecular methods

Total genomic DNA was extracted from livers and spleens with a QIAamp Tissue kit (QIAGEN, Hilden, Germany), as described by the manufacturer. Samples were handled under sterile conditions to avoid the risk of cross-contamination.

Samples were screened for the presence of *Bartonella* species DNA by the use of a real-time (RT) quantitative PCR targeting the ITS gene. Positive samples at screening were further studied by PCR amplification and DNA sequencing of the genes encoding the 16S–23S rDNA intergenic spacer (ITS), citrate synthase (*gltA*), β-subunit of the RNA polymerase (*rpoB*) using previously described primers and conditions 5. Similarity rates between all the identified *Bartonella* species were determined for each gene to assess the taxonomic position of *Bartonella* isolates at the species levels. Phylogenetic relationships among the studied bartonellae were inferred from sequence alignments of each gene and from concatenated gene sequences using the maximum parsimony and neighbour-joining methods within the MEGA version 3.1 software package (Megasoftware, Tempe, AZ, USA).

## Results

We examined 568 tissue samples (311 livers and 257 spleens) from 310 rodents and one tree shrew (see Table 1). One hundred and twenty-eight tissue samples (22.5%), corresponding to 79 rodents (25.5%), were positive for *Bartonella* by RT-PCR. Positive and negative controls showed expected results in all tests. Using standard ITS PCR and sequencing we identified the presence of five *Bartonella* species, including *B. phoceensis*, *B. elizabethae*, *B. tribocorum*, and two new *Bartonella* species that we named Lao/Nh1 and Lao/Nh2, in three different regions of Laos (Table 1). Lao/Nh1 shared less than 96.0% sequence similarity for a 327-bp *gltA* fragment of the validated *Bartonella* spp. and less than 95.4% for a 825-bp *rpoB* fragment. Lao/Nh2 shared less than 95.4% for a 825-bp *rpoB* fragment of the validated *Bartonella* spp. and a 327-bp *gltA* fragment with 97.8% similarity with *B. tribocorum*.

**Table 1:** Species of rodents and *Bartonella* species detected at different collection sites in Lao PDR

Location, % rodents with *Bartonellae*	Rodent species	No. of spleen/liver	*Bartonella*	% all rodents with *Bartonella*
Vientiane City, 15.5%	*Rattus rattus*	79/77	*B. phoceensis* (1) and *B. eizabethae* (2), *B. tribocorum* (2) and Lao/Nh2 (3)	10.1%
	*R. exulans*	23/23	*B. elizabethae* (1), *B. phoceensis* (1) and *B. tribocorum* (5)	30.4%
	*Mus cervicolor*	6/6	Lao/Nh2 (1)	16.7%
	*Mus caroli*	2/2	*B. phoceensis* (1)	50%
Luang Prabang, 11.2%	*R. rattus*	141/139	*B. elizabethae* (4), *B. phocensis* (8), Lao/Nh2 (8), Lao/Nh1 (1)	9.1%
	*Cannomys badius*	2	Lao/Nh2 (1)	50%
	*B. indica*	2	0	0
	*M. caroli*	1	0	0
Champasak, 12.4%	*M. cervicolor*	64/65	0	0
	*R. rattus*	1/1	0	0
	*R. exulans*	56/56	*B. phoceensis* (2), *B. elizabethae* (1), *B. tribocorum* (1) and Lao/Nh2 (6)	17.9%
	*Petinomys phayrei*	2/2	0	0
Luang Nam Tha, 19.2%	*R. rattus*	160/159	*B. phoceensis* (11), *B. elizabathae* (2), *B. tribocorum* (3), Lao/Nh1 (5), Lao/Nh2 (11)	20.1%
	*R. argentiventer*	1/1	0	0
	*Rhizomys sumatrensis*	2/3	0	0
	*Callosciurus erythraeus*	2/2	0	0
	*Dremomys rufigenis*	1/1	0	0
	*B. savilei*	1/1	0	0
	*R. exulans*	1/1	0	0

## Discussion

Comparison of DNA sequences has been the most commonly used approach for *Bartonella* species identification as the members of the genus *Bartonella* are fastidious bacteria that possess few phenotypic markers that are useful for species delineation. We used the *gltA*, the *rpoB* and the ITS genes to determine the taxonomic status of *Bartonella* strains from Laos as these genes have good discriminating power 5. According to current molecular criteria 5, Lao/Nh1 and Lao/Nh2 could be defined as new species. Using concatenation of the sequences obtained, Lao/Nh1 clustered with *B. birtlesii* and *B. taylorii* whereas Lao/Nh2 clustered with *B. tribocorum*. Finally, Lao/Nh1 was detected only in *Rattus rattus* collected from Luangnatha Province and Luang Prabang. It is yet unknown whether these *Bartonella* species strains from Laos are human pathogens and this should be investigated.

To the best of our knowledge, this is the first study of the prevalence of *Bartonella* species in rodents in Lao PDR. We found a high prevalence of *Bartonella* spp. (25.5%), including *B. elizabethae*, *B. tribocorum* and *B. phoceensis*. *B. elizabethae* has been already isolated in small mammals from Bangladesh 2 and *B. tribocorum* in rodent fleas from China 6. Interestingly, *B. tamiae*, a newly recognised pathogen isolated from three human patients from Thailand 3, was not detected in Lao rodents. Concerning *B. phoceensis*, it is its first molecular detection in this part of the world.

*Bartonella* spp. were identified in rodents derived from both agrarian and urban environments. Rodents are abundant in both contexts in Laos, hence a large proportion of the human population lives in close contact with rodents. Accordingly, it is possible that some unknown diseases in Laos may be caused by *Bartonella* spp. Further investigations are warranted in order to isolate these new *Bartonella* spp. and to determine if they can cause any clinical manifestations.
